# Semi-Automated Analysis of Diaphragmatic Motion with Dynamic Magnetic Resonance Imaging in Healthy Controls and Non-Ambulant Subjects with Duchenne Muscular Dystrophy

**DOI:** 10.3389/fneur.2018.00009

**Published:** 2018-01-26

**Authors:** Courtney A. Bishop, Valeria Ricotti, Christopher D. J. Sinclair, Matthew R. B. Evans, Jordan W. Butler, Jasper M. Morrow, Michael G. Hanna, Paul M. Matthews, Tarek A. Yousry, Francesco Muntoni, John S. Thornton, Rexford D. Newbould, Robert L. Janiczek

**Affiliations:** ^1^Imanova Limited, Hammersmith Hospital, London, United Kingdom; ^2^Dubowitz Neuromuscular Centre, UCL Great Ormond Street Institute of Child Health, London, United Kingdom; ^3^MRC Centre for Neuromuscular Diseases, UCL Institute of Neurology, London, United Kingdom; ^4^Neuroradiological Academic Unit, UCL Institute of Neurology, London, United Kingdom; ^5^Division of Brain Sciences, Centre for Neurotechnology, UK Dementia Research Institute, Imperial College London, London, United Kingdom; ^6^GlaxoSmithKline plc., Stevenage, United Kingdom

**Keywords:** Duchenne, Duchenne muscular dystrophy, Magnetic Resonance Imaging, dynamic, respiration, diaphragm, non-ambulatory

## Abstract

Subjects with Duchenne Muscular Dystrophy (DMD) suffer from progressive muscle damage leading to diaphragmatic weakness that ultimately requires ventilation. Emerging treatments have generated interest in better characterizing the natural history of respiratory impairment in DMD and responses to therapy. Dynamic (cine) Magnetic Resonance Imaging (MRI) may provide a more sensitive measure of diaphragm function in DMD than the commonly used spirometry. This study presents an analysis pipeline for measuring parameters of diaphragmatic motion from dynamic MRI and its application to investigate MRI measures of respiratory function in both healthy controls and non-ambulant DMD boys. We scanned 13 non-ambulant DMD boys and 10 age-matched healthy male volunteers at baseline, with a subset (*n* = 10, 10, 8) of the DMD subjects also assessed 3, 6, and 12 months later. Spirometry-derived metrics including forced vital capacity were recorded. The MRI-derived measures included the lung cross-sectional area (CSA), the anterior, central, and posterior lung lengths in the sagittal imaging plane, and the diaphragm length over the time-course of the dynamic MRI. Regression analyses demonstrated strong linear correlations between lung CSA and the length measures over the respiratory cycle, with a reduction of these correlations in DMD, and diaphragmatic motions that contribute less efficiently to changing lung capacity in DMD. MRI measures of pulmonary function were reduced in DMD, controlling for height differences between the groups: at maximal inhalation, the maximum CSA and the total distance of motion of the diaphragm were 45% and 37% smaller. MRI measures of pulmonary function were correlated with spirometry data and showed relationships with disease progression surrogates of age and months non-ambulatory, suggesting that they provide clinically meaningful information. Changes in the MRI measures over 12 months were consistent with weakening of diaphragmatic and inter-costal muscles and progressive diaphragm dysfunction. In contrast, longitudinal changes were not seen in conventional spirometry measures during the same period. Dynamic MRI measures of thoracic muscle and pulmonary function are, therefore, believed to detect meaningful differences between healthy controls and DMD and may be sensitive to changes in function over relatively short periods of follow-up in non-ambulant boys with DMD.

## Nomenclature

**Table:** 

ANT	The 1-D lung length at the anterior chest wall margin
CNT	The 1-D lung length at the (central) tracheal bifurcation point
CSA	Cross-sectional area
DIA	Diaphragm length
DMD	Duchenne Muscular Dystrophy
FVC	Forced vital capacity
LOA	Loss of ambulation
MEP	Maximal expiratory pressure
MIP	Maximal inspiratory pressure
MRI	Magnetic Resonance Imaging
PCF	Peak cough flow
PEF	Peak expiratory flow
PST	The 1-D lung length at the posterior chest wall margin
%Pred	FVC as percentage of predicted normal values
*R*	Correlation coefficient of the linear regression
*S*	Slope of the linear regression
TDM	Total distance of motion of the diaphragm

## Introduction

Duchenne Muscular Dystrophy (DMD) is a highly debilitating X-linked recessive disorder affecting 1 in 5,000 male births (1). Mutations in the *DMD* gene preclude expression of the sarcolemmal protein dystrophin, which is found primarily in skeletal and cardiac muscle. This leads to progressive muscle damage, loss of function, and loss of ambulation (LOA) by their mid-teens (2). DMD patients can also suffer from respiratory failure, ultimately requiring ventilation (3, 4).

Until very recently, glucocorticoid therapy was the only pharmacological intervention proven to delay disease progression in muscles (5); however, a phosphorodiamidate morpholino antisense oligonucleotide (PMO) that modulates splicing to restore semi-functional dystrophin has now received conditional approval by the Food and Drug Administration to treat DMD (6) and a drug to induce read-through nonsense mutations has been approved within the European Union Member States (7). Outcome measures in clinical trials currently rely on the assessment of motor function, such as the 6-minute-walk test (6-MWT), which has been utilized as a primary outcome end-point in phase II–III clinical trials (7–9).

Driven by the emerging treatments, there is rapidly growing interest in better understanding the natural history of respiratory function in DMD and response to therapy. This is currently tested using spirometry. Recent studies have described the spirometry measure of forced vital capacity (FVC) as a percentage of predicted normal values (FVC% predicted) in both ambulant and non-ambulant DMD (10, 11), and used this measure to show the potential benefit of glucocorticoid therapy in stabilizing pulmonary function. Respiratory function could therefore offer a potential outcome measure in clinical trials, especially in the non-ambulant population who cannot undertake tests such as the 6-MWT, and in most cases, are excluded from clinical trials. Indeed, spirometry was the primary outcome measure of a recently completed trial investigating a drug in non-ambulant DMD patients (12). However, spirometry does have some challenges especially in the DMD population, since the readings can be highly variable, rely on subject motivation and cooperation, as well as an ability to comprehend and follow specific instructions. These factors can provide a challenge for DMD individuals not only in view of their young age but also the neurobehavioural and cognitive involvement that characterizes at least 30% of the population (13).

The aim of our study was to explore whether a novel image analysis of dynamic (cine) thoracic Magnetic Resonance Imaging (MRI) can be used to provide an adjunctive and more sensitive/objective measure of pulmonary function and diaphragm mobility in DMD than conventional spirometry. Exploratory MRI endpoints derived from temporal profiles of lung and diaphragm measurements were investigated in both healthy controls and non-ambulant DMD boys. To enable this, we extended previous and similar work that has used proton (^1^H) MRI to assess pulmonary function in healthy volunteers (14–18).

## Patients and Methods

### Subjects

Thirteen non-ambulant DMD boys (mean age 13.2 ± 2.1 years) and 10 age- and gender-matched healthy volunteers [mean age 14.6 ± 1.3 years; *P* = 0.083 (two-tailed *t*-test)] were recruited and scanned at baseline, with a subset (*n* = 10, 10, 8) of the DMD subjects also assessed at follow-up visits 3, 6, and 12 months later. All patients had genetically confirmed DMD and were on glucocorticoids. The withdrawals at follow-up were due to the burden of the frequent hospital visits, which were challenging to sustain in the absence of therapeutic intervention. Approval from the Brighton & Sussex Research National Ethics Committee was obtained for this study, which was performed in compliance with the Declaration of Helsinki. Written informed consent for participation in the study was obtained from a representative parent or caregiver and assent forms were signed by the DMD boys and healthy volunteers. The study was sponsored by University College London.

### Clinical Assessment

Each DMD subject was assessed by the following measurements: weight and height; spirometry and peak cough flow (PCF) using a Vitalograph Pneumotrac 6800; and record of time of LOA in months. The spirometry parameters recorded (as the best of three attempts) were FVC and FVC% predicted (%Pred), PCF, peak expiratory flow (PEF), maximal inspiratory pressure (MIP), and maximal expiratory pressure (MEP). FVC and %Pred were typically recorded in the standard sitting position as per clinical practice. Weight and height of the healthy volunteers were recorded for comparison.

### MRI Data

**Figure 1:**
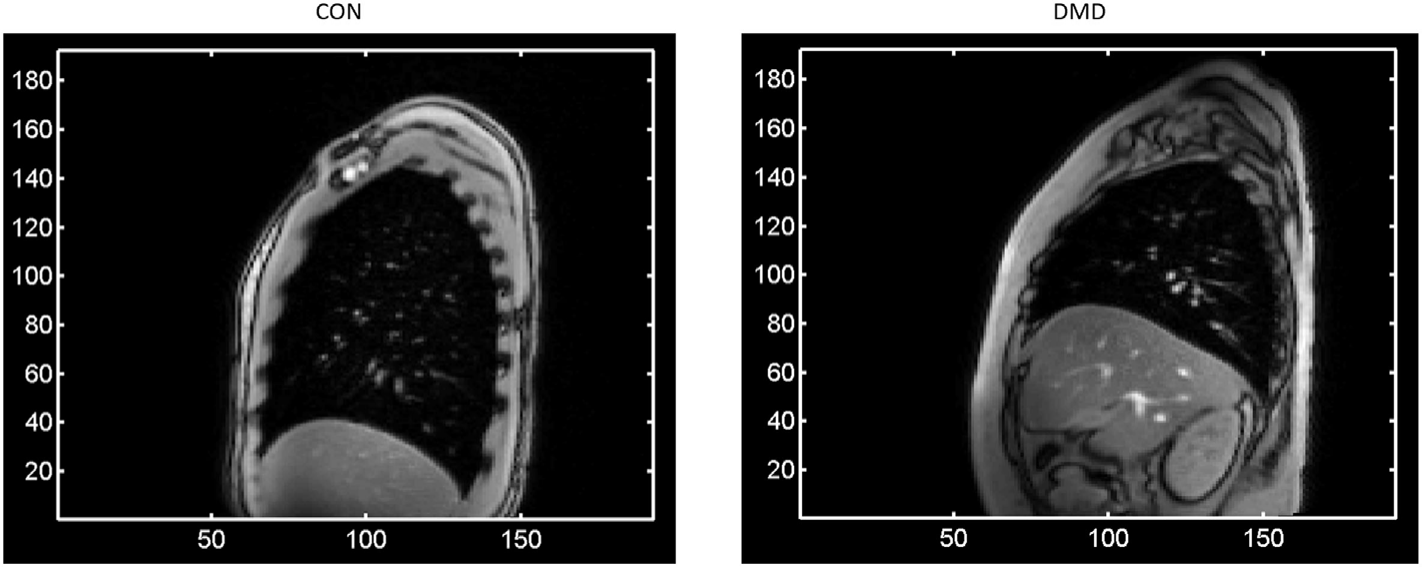
A single frame of the right sagittal dynamic Magnetic Resonance Imaging data for two representative subjects at baseline—one from each group [left: a control; right: a Duchenne Muscular Dystrophy (DMD) patient]—showing at full lung capacity the much larger lung cross-sectional area for the control compared to the DMD subject, as well as the typical levels of signal-to-noise and the observed image features such as the contrast at the periphery of the lung. The axes represent pixel coordinates, and the image field-of-view was the same in each case.

Dynamic (cine) MRI data were acquired at each visit in each orthogonal imaging plane (axial, sagittal, coronal) on a Siemens Skyra 3T scanner, separately for both free and deep breathing conditions in DMD subjects and healthy controls. For each imaging plane, the MRI acquisition involved a single slice imaged over time (i.e., dynamic imaging) to allow monitoring of the respiratory activity of the subjects over multiple breath cycles. For the deep (max) breathing condition, subjects were instructed to breathe in and out deeply three times upon hearing the scanner noise. There were two imaging planes (left and right) for the sagittal acquisitions, with the single slice positioning located at the midline (mid-clavicular level) of each lung. Right sagittal images for two representative subjects (one from each group) are shown in Figure 1. This work covers analysis of the right sagittal imaging data during the deep breathing condition.

The right sagittal dynamic MRI data were acquired using generalized autocalibrating partially parallel acquisitions (19) parallel imaging (*R* = 2 acceleration factor) and 6/8 phase partial Fourier acquisition in a gradient recalled mode with the following parameters: repetition time of 3 ms, echo time of 1.39 ms, flip angle of 8°, 384 mm × 384 mm field of view, in-plane image resolution of 2.0 mm × 2.0 mm, and 5 mm slice thickness. Typically 60 frames were sampled, with a time of 0.5 s to acquire each image slice.

### Image Analysis

**Figure 2:**
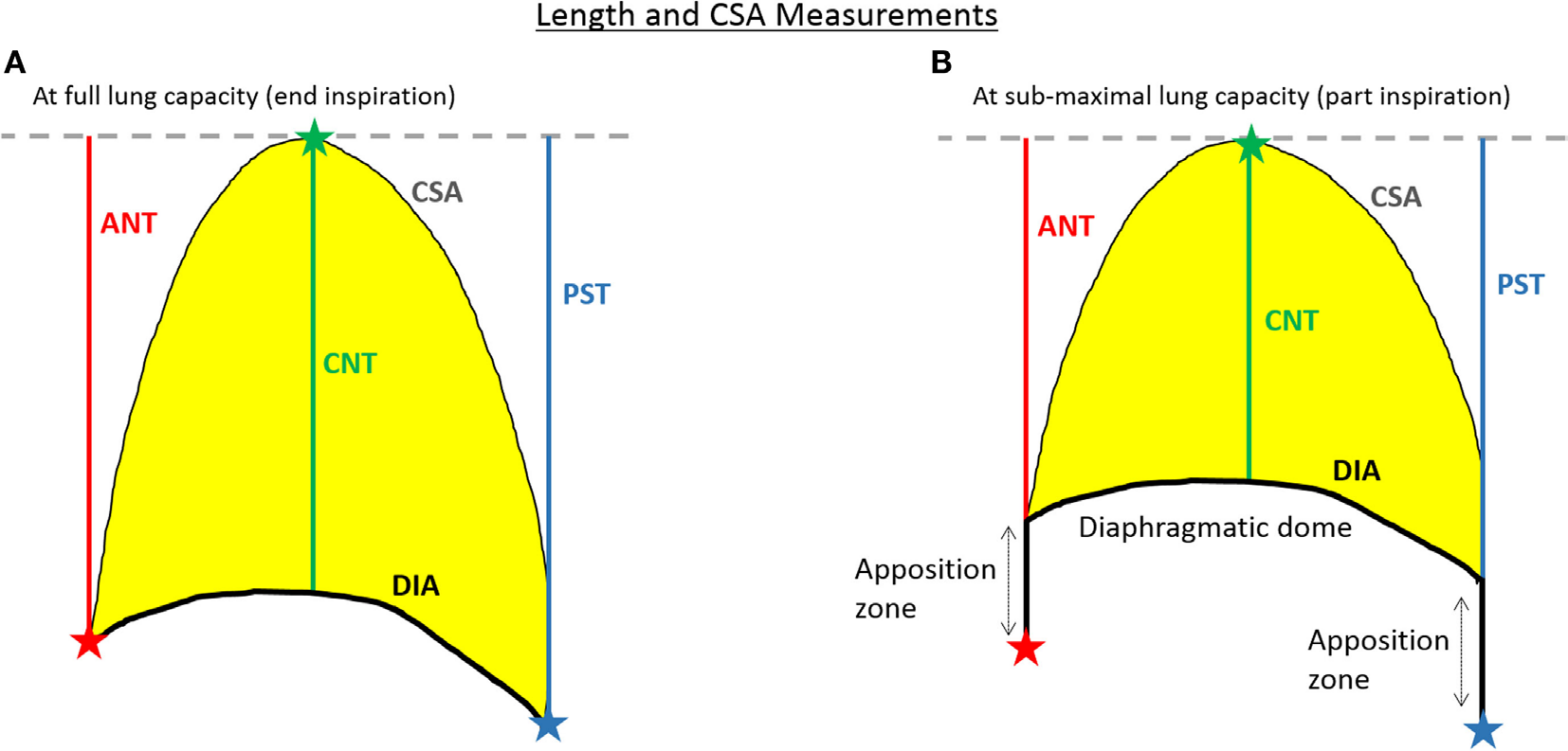
A schematic of the extracted lung and diaphragm measures at both full lung capacity **(A)** and at reduced lung capacity **(B)**. At full lung capacity (i.e., end inspiration) the diaphragm length (DIA) is the length across the diaphragmatic dome from the anterior to the posterior chest wall margin, while at sub-maximal lung capacity (i.e., part inspiration) the full DIA is computed as the length over the diaphragmatic dome plus the lengths along the zones of apposition, without changing the points of attachment of the diaphragm at the anterior and posterior chest wall margins. The total distance of motion (TDM) of the diaphragm is computed as the sum of the ANT, CNT, and PST lengths. The cross-sectional area (CSA) is the area inside the lung contour (coloured yellow).

An analysis pipeline was developed in MATLAB (http://uk.mathworks.com/products/matlab/) to extract temporally varying profiles of lung and diaphragm measures from dynamic MRI data for the investigation of exploratory endpoints related to pulmonary function. Details of the image analysis pipeline are given in Section S1 in Data Sheet S1 and Figure S1 in Supplementary Material, together with a schematic representation of the process. Figure 2 illustrates the lung and diaphragm measures extracted from every frame of the dynamic MRI data. Broadly speaking, after automatic segmentation of the lung cross-sectional area (CSA) on each frame, three 1-D lung length measures are computed—one at the anterior chest wall margin, one at the tracheal bifurcation point, and one at the posterior chest wall margin (denoted ANT, CNT, and PST, respectively), as well as the diaphragm length (DIA). The lung length measurements, which reflect diaphragm movement, are summed together to provide an estimate of the total distance of motion (TDM) of the diaphragm (16, 18). The DIA is computed as described in the caption of Figure 2.

**Figure 3:**
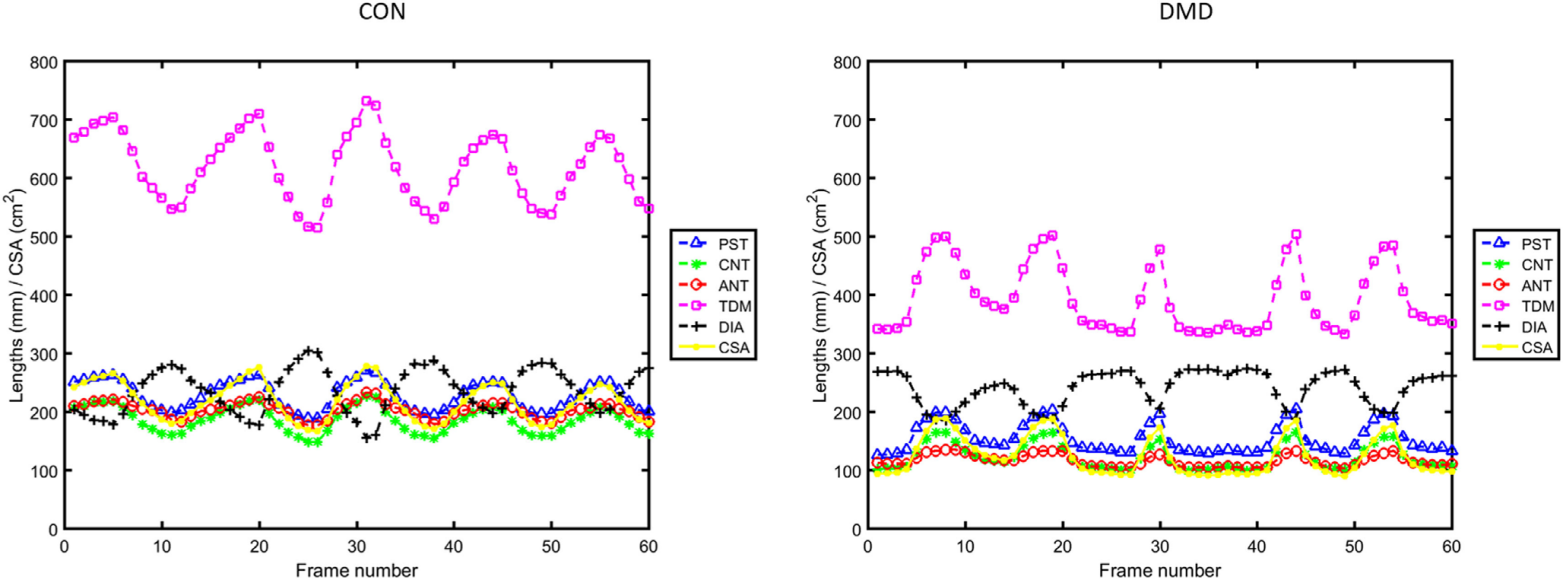
Plots of the temporal variation in lung and diaphragm measurements across the full duration of the dynamic Magnetic Resonance Imaging data for the same two representative subjects as in Figure 1 [left: a control; right: a Duchenne muscular dystrophy (DMD) patient].

For MRI data such as presented here, with relatively good signal-to-noise ratio, the image analysis pipeline can automatically proceed without interruption using default parameters, pausing only to request user input of three initialization points on a single frame for locating the anatomical landmarks (see Section S1 in Data Sheet S1 and Figure S1 in Supplementary Material). For this study, the three initialization points for each subject were defined by a single observer (Courtney A. Bishop)—a Ph.D. with 9 years of clinical MRI analysis experience and the developer of the presented image analysis pipeline. After image processing, the in-house MATLAB program generates plots of the temporal variation in CSA and length measures (e.g., Figure 3), a movie showing the lung segmentation and length measures on every frame of the dynamic MRI (see Video S1 in Supplementary Material), and a table containing the full complement of extracted *summary measures* (described in the Section “Outcome Measures”).

### Outcome Measures

The collinearity of diaphragm and lung length measures with the CSA (Figure 3) across the respiratory cycles was explored quantitatively using regression analysis [similar to the length versus lung volume relationships presented for healthy subjects in Ref. (16)]. The slope (*S*), *y*-axis intercept (*C*), correlation coefficient (*R*), and *P*-value of the linear regressions were computed. From the temporally varying profiles of lung and diaphragm parameters, we extract descriptive statistical measures that are collectively referred to herein as the diaphragm motion *summary measures*: the minimum, maximum, delta (=maximum–minimum), mean, and standard deviation (SD) of each measure (CSA, ANT, CNT, PST, TDM, and DIA) for all subjects. For the baseline DMD data, the relationships between the diaphragm motion summary measures and the spirometry measures (of FVC sitting and %Pred, PEF, PCF, MIP, and MEP) were explored with the Pearson correlation coefficient, with a nominal *P*-value < 0.05 considered statistically significant. The baseline relationship between FVC sitting and FVC supine was determined *via* a non-parametric Wilcoxon matched pairs test. Longitudinal change in both the spirometry data and the above-mentioned outcome measures (the linear regression parameters and the MRI summary measures) were explored using mixed modeling across visit (nominal, fixed factor) with subject as a random factor. Additionally, the longitudinal relationships with the continuous disease progression surrogates of age and months of non-ambulation (the dependent variables) were explored using a mixed model approach with subject as a random factor and the MRI-derived summary measures or the spirometry data as the covariate of interest. Due to the exploratory nature of this work, the *P*-values reported throughout are without correction for multiple comparisons.

#### Intra-and Inter-Observer Variability Testing

**Figure 4:**
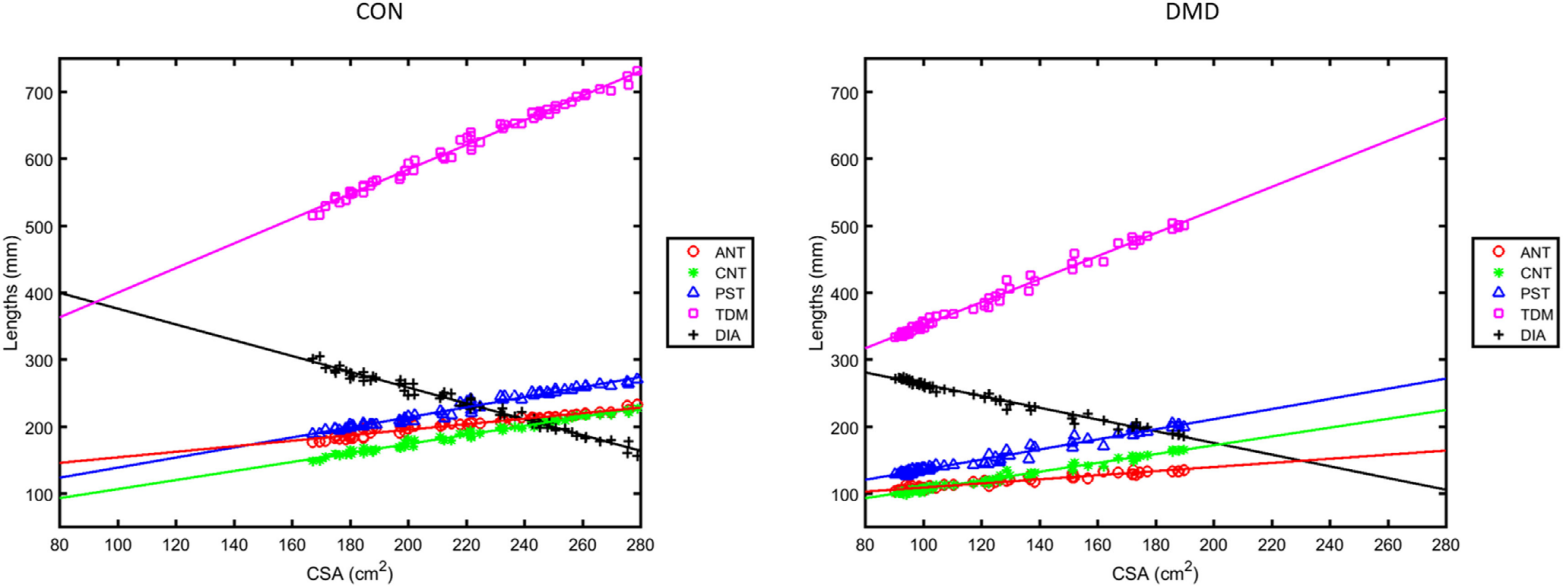
Regression plots of the lengths ANT, CNT, PST, total distance of motion (TDM) and diaphragm length (DIA) versus lung cross-sectional area (CSA) measured from the sagittal images during deep breathing for the same two representative subjects as in Figures 1 and 3 [left: a control; right: a Duchenne muscular dystrophy (DMD) patient].

To test the influence of the three initialization points on the outcome summary measures, two image analysts (Courtney A. Bishop—original analyst—and Graham E. Searle—second observer, Ph.D. with 11 years of experience as a medical image analyst for both PET and MRI) each defined five sets of initialization points on six test subjects. The six test subjects consisted of three DMD boys and three healthy controls: the two representative subjects (one DMD and one control) featured in the manuscript (Figures 1, 3 and 4) plus two additional DMD boys and two additional healthy controls selected at random. For training purposes, Courtney A. Bishop provided Graham E. Searle with a verbal description and visual demonstration of landmark initialization on an independent, randomly selected sample subject prior to Graham E. Searle defining the initialization points. For each set of initialization points, the temporally varying lung and DIA measures were computed, and from these, the corresponding diaphragm motion summary measures. For the min ANT, min CNT, min PST, and min DIA lengths, the percentage coefficients of variation were calculated for each individual user and each test subject to inform on intra-observer variability, and a two-sided Wilcoxon rank sum test was performed to test for inter-observer variability (*N* = 24).

## Results

### Clinical Assessments

Two-sample *t*-tests (two-tailed) showed that the mean baseline weight for the *n* = 13 DMD boys (59.6 ± 17.0 kg) did not differ significantly from that of the healthy volunteers (60.2 ± 13.0 kg; *P* = 0.93), but the DMD boys were shorter (1.48 ± 0.09 m) and had an increased body mass index (26.9 ± 5.9) compared to the controls [1.66 ± 0.12 m (*P* < 0.001) and 21.5 ± 2.3 (*P* = 0.012), respectively]. Mean duration without ambulation for the DMD boys was 20.9 ± 12.6 months at baseline. Height was unchanged for all of the DMD boys over the 12-month study duration.

### Spirometry Measures

Baseline mean (and SD) for the spirometry measures of FVC, %Pred, PEF, PCF, MIP, and MEP for DMD subjects are given in Table 1, together with their percentage change from baseline at the follow-up visits 3, 6, and 12 months later. At baseline, a Wilcoxon matched pairs test (*n* = 7) did not suggest differences between FVC sitting and FVC supine. Mixed modeling of the longitudinal FVC data (*n* = 29) shows that the two FVC measures are significantly related (*t* = 2.20, *P* = 0.044). Since sitting spirometry is the most commonly used measure in clinical settings, all of the remaining analyses with FVC (e.g., versus MRI-derived metrics and the disease progression markers of age and months of non-ambulation) were done with the values obtained in a sitting position.

**Table 1:** Mean (SD) of the spirometry and peak cough flow (PCF) metrics at baseline (first column) and their percentage change from baseline (columns 2–4).

	Baseline	3-month change, %	6-month change, %	12-month change, %
FVC (L)	2.09 (0.50); *N* = 12	−9.99 (16.7); *N* = 9	−2.14 (11.0); *N* = 9	−1.96 (17.0); *N* = 7
%Pred	71.2 (12.5); *N* = 10	−2.81 (8.66); *N* = 6	1.56 (6.80); *N* = 7	−6.37 (14.4); *N* = 6
PEF (L/min)	3.98 (1.06); *N* = 12	0.29 (30.5); *N* = 9	13.0 (30.5); *N* = 9	−16.8 (33.0); *N* = 7*
PCF (L/s)	3.89 (1.31); *N* = 11	−13.8 (22.1); *N* = 8	21.8 (37.6); *N* = 8	−6.92 (23.3); *N* = 6
MIP (cm H_2_O)	46.6 (16.1); *N* = 12	26.9 (43.8); *N* = 9	13.3 (29.7); *N* = 9	32.8 (56.2); *N* = 5
MEP (cm H_2_O)	36.9 (18.1); *N* = 12	111.1 (158.2); *N* = 9	127.8 (175.6); *N* = 9	191.9 (233.9); *N* = 5

*FVC, forced vital capacity; %Pred, FVC as percentage of predicted normal values; PEF, peak expiratory flow; MIP, maximal inspiratory pressure; MEP, maximal expiratory pressure; L, liters*.

*P ≤ 0.05 for post hoc pairwise comparisons of absolute measurements between visits, two-tailed.

### The Linearity of Chest Wall Motions and Evidence of Dyssynchrony in DMD

**Figure 5:**
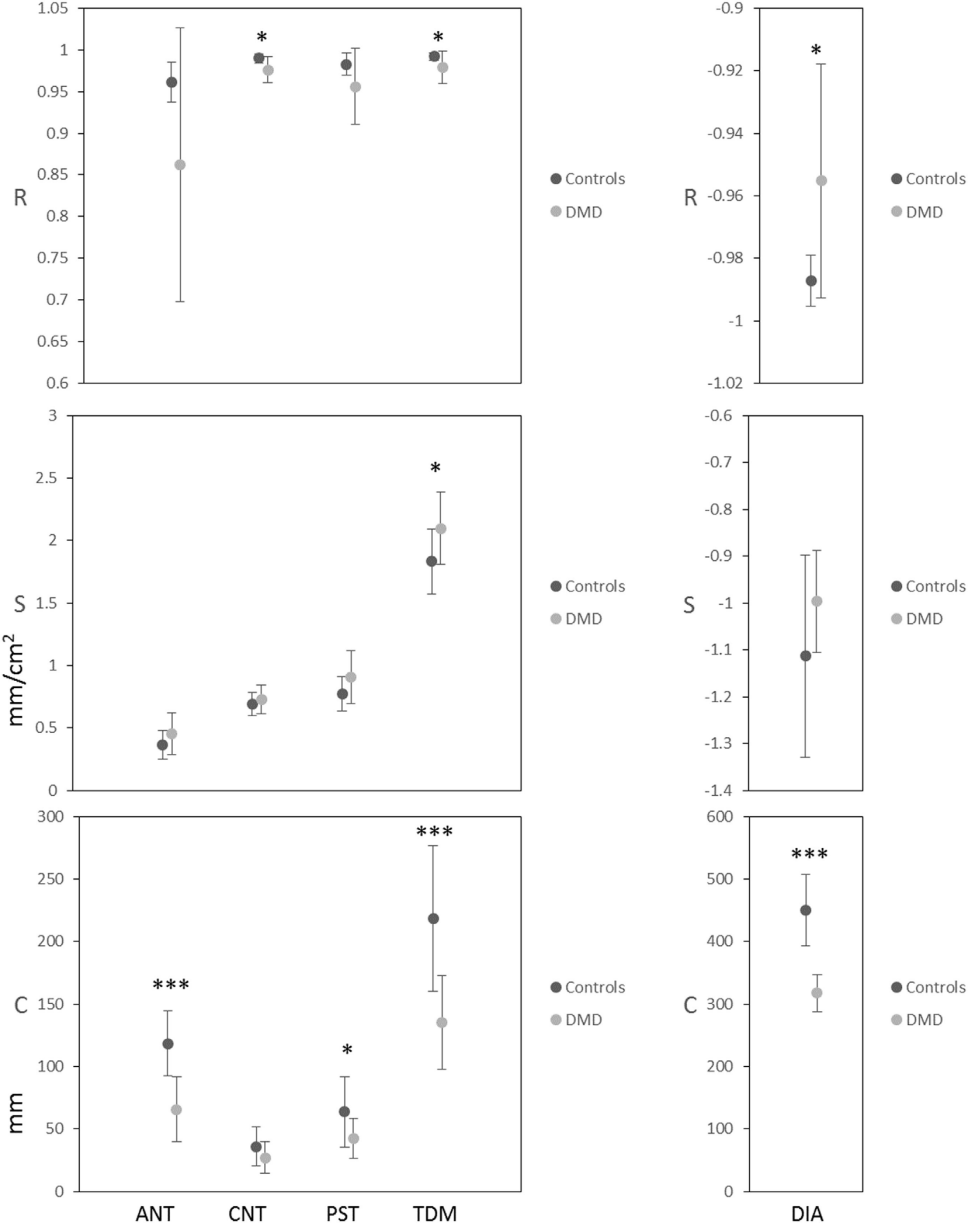
Mean and SD of the correlation coefficient (*R*: top row), the slope (*S*: middle row) and the *y*-axis intercept (*C*: bottom row) of the linear regressions for lung cross-sectional area (CSA) versus each of the length measures ANT, CNT, PST, total distance of motion (TDM) and diaphragm length at baseline. *P*-values for the two-sample *t*-tests, comparing controls to Duchenne muscular dystrophy (DMD), are indicated as ***P ≤ 0.001, **P ≤ 0.01, *P ≤ 0.05.

Plots from the linear regression analysis—investigating the relationship between the change in length measures and the change in CSA—are shown in Figure 4 for the same two representative subjects as in Figures 1 and 3. The mean and SD of the linear regression parameters (*R, S*, and *C*) for the control and DMD groups at baseline are given in Figure 5. All of the length measures correlated well with the lung CSA, as indicated by the high *R* (Figure 5: top row). If *R* is high, the *S* of the linear regression line between any given length measure and the lung CSA represents the contribution of the change in that length measure to a unit change in CSA. In this work, the *C* represents the lengths in a hypothetical scenario where the CSA is equal to 0, enabling cross-subject comparison in cohorts with significantly different CSA at end expiration. The range of CSA over which these linear relationships were modeled and demonstrated to be a good fit, was 102.9–378.5 cm^2^ for the controls and 43.4–189.8 cm^2^ for DMD.

At baseline, *R* values were high (Figure 5) and the *P*-value of all regression lines were much less than 0.05 so the relationships were considered to be sufficiently linear in both groups. Controls did, however, have significantly higher *R* (linearity/synchrony) for the CNT length, the TDM, and the DIA length compared to the DMD group (*P* = 0.015, 0.049, and 0.015, respectively). The *S* of the CSA versus TDM regression lines were significantly higher in the DMD group than the controls (*P* = 0.035), indicating a larger change in TDM in DMD patients per unit change in CSA. The *C* revealed the greatest differences between the groups at baseline: significantly higher *C* was found in the ANT (*P* < 0.001), the PST (*P* = 0.030), the TDM (*P* < 0.001), and the DIA (*P* < 0.001) of the controls compared to DMD, showing that the end-expiratory lung and diaphragm lengths were longer in the controls.

The mixed modeling revealed no overriding longitudinal change (main effect of visit) for the regression parameters, although there was a trend for longitudinal change in the *C* for ANT length [*F*(3,18) = 2.67, *P* = 0.078] and *post hoc* pairwise comparisons suggested that this parameter may be reduced as early as the 3-month follow-up (*P* = 0.029, two-tailed).

### Diaphragm Motion Summary Measures

#### Intra-and Inter-Observer Variability

There was clear agreement of the summary measure lengths calculated from the repeated initialization points within and between observers (Figure S2 in Supplementary Material). For the min ANT, min CNT, min PST, and min DIA lengths, the percentage coefficients of variation for each individual user and each test subject (Section S2 in Data Sheet S1 in Supplementary Material) ranged from 0 to 1.48% for observer 1 (Courtney A. Bishop) and from 0 to 2.50% for observer 2 (Graham E. Searle), indicating extremely low levels of intra-observer variability of the analysis method. A two-sided Wilcoxon rank sum test indicated no significant difference in the %CV between observers (*N* = 24, *Z* = −0.775, *P* = 0.438), so there was no detectable inter-observer variability in the method.

#### Cross-Sectional Group Differences and Relationships with Spirometry in DMD

Table 2 presents the baseline group means and SD of the MRI summary measures (min, max, etc.) extracted from the temporal profiles of the length and CSA measurements. The DMD max CSA was 45% smaller than the controls, while the delta CSA and max TDM were 40% smaller and 37% smaller, respectively. Highly significant group differences (indicated by the asterisks in Table 2) were observed at baseline for most of the summary measures even after accounting for the potentially confounding differences in height between the controls and the DMD boys. Exceptions were the delta ANT length, the max DIA and the mean DIA length, where no baseline group differences were found.

**Table 2:** Group mean (SD) of the summary measures at baseline: the minimum (min), maximum (max), delta (max–min) and the mean of the CSA, anterior (ANT), central (CNT), and posterior (PST) lung lengths, total distance of motion (TDM) of the diaphragm, and diaphragm length (DIA).

	Min		Max		Delta		Mean	
	CON	DMD	CON	DMD	CON	DMD	CON	DMD
CSA (cm^2^)	157.3 (35.5)	82.4 (14.6)***	259.9 (50.9)	143.0 (24.0)***	102.6 (25.6)	60.7 (21.2)***	205.8 (47.5)	107.0 (14.8)*
ANT (mm)	172.6 (24.9)	96.9 (20.1)***	211.0 (27.3)	129.2 (21.1)***	38.4 (9.9)	32.3 (11.1)	191.4 (26.4)	113.4 (19.5)***
CNT (mm)	146.2 (27.3)	86.8 (11.2)***	217.3 (35.4)	130.5 (13.2)***	71.1 (16.4)	43.8 (11.2)**	178.1 (34.2)	104.6 (10.4)***
PST (mm)	182.6 (27.7)	115.7 (11.1)***	258.0 (31.3)	171.2 (20.5)***	75.5 (14.0)	55.5 (19.4)	219.3 (34.0)	138.8 (11.5)***
TDM (mm)	501.9 (77.8)	302.2 (33.9)***	685.1 (88.5)	429.8 (39.9)***	183.2 (32.8)	127.6 (37.2)*	588.9 (92.5)	356.7 (29.2)***
DIA (mm)	161.9 (7.4)	174.5 (18.4)*	276.5 (25.5)	239.0 (31.6)	114.6 (25.6)	64.5 (17.6)**	224.3 (17.1)	211.8 (24.9)

***P ≤ 0.001, **P ≤ 0.01, *P ≤ 0.05 two-tailed, height as covariate.

*N = 10 for healthy controls (CON), N = 13 for Duchenne muscular dystrophy (DMD) boys*.

There was good correlation of several summary measures with the spirometry measures of FVC (sitting), %Pred, and PEF recorded for the DMD group at baseline (Table 3). The strongest relationships, with lowest *P*-values and highest Pearson correlation coefficients, were FVC versus max TDM (*R* = 0.777, *P* = 0.0029) and %Pred versus mean TDM (*R* = 0.795, *P* = 0.0060).

**Table 3:** Pearson correlation coefficients for the statistically significant linear relationships between the Magnetic Resonance Imaging summary measures and the spirometry data at baseline.

	DMD (visit 1, *N* = 13)											
	Max CSA	Delta CSA	Mean CSA	Max TDM	Delta TDM	Mean TDM	Delta ANT	Max CNT	Mean CNT	Max PST	Delta PST	Mean PST
Forced vital capacity (*N* = 12)	0.696**	0.609*	0.647*	0.777**	0.617*	0.666*	–	–	–	0.654*	0.619*	0.639*
%Pred (*N* = 10)	–	–	–	0.715*	–	0.795**	–	0.725*	0.701*	–	–	–
Peak expiratory flow (*N* = 12)	–	0.624*	–	–	0.673*	–	0.623*	–	–	–	0.586*	–

***P ≤ 0.001, **P ≤ 0.01, *P ≤ 0.05.

*DMD, Duchenne muscular dystrophy; CSA, cross-sectional area; TDM, total distance of motion*.

#### Longitudinal Findings in DMD and Relationships with Disease Progression Metrics

**Figure 6:**
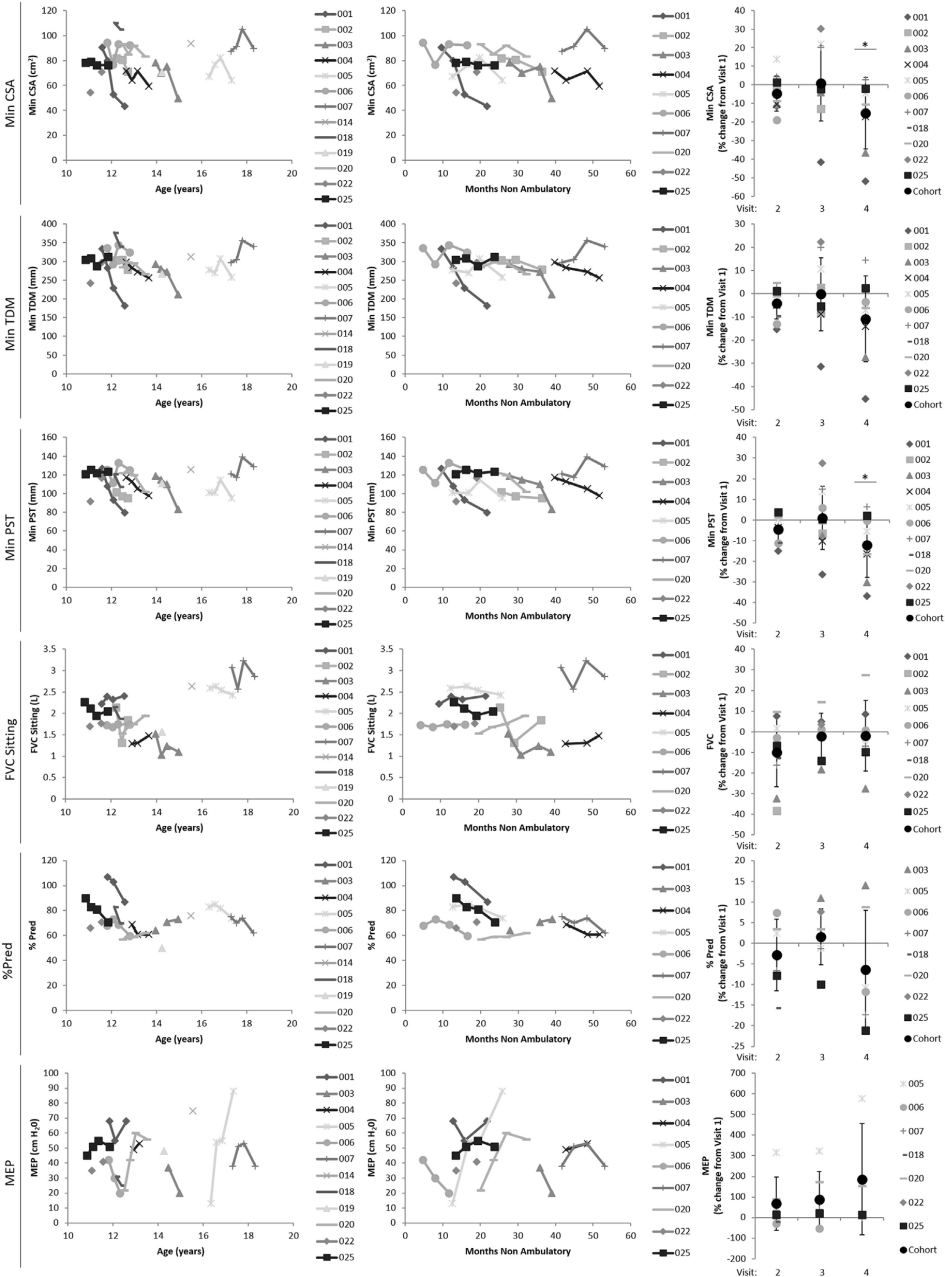
Plots showing how a subset of the Magnetic Resonance Imaging-derived summary measures (min CSA, top row; min TDM, second row; max TDM, third row) and spirometry metrics (FVC, fourth row; %Pred, fifth row; and MEP, last row) relate to the disease progression surrogates of age (left plots) and months non-ambulatory (middle plots), as well as follow-up visit (right plots). Min CSA, minimum lung cross-sectional area; min/max TDM, minimum/maximum total distance of motion (TDM) of the diaphragm; FVC, forced vital capacity; %Pred, FVC as a percentage of predicted normal values; MEP, maximal expiratory pressure.

The mean percentage change from baseline for the CSA, TDM, and DIA summary measures are given in Table 4 for the DMD longitudinal data. Figure 6 shows how a subset of the summary measures and the spirometry metrics relate to the disease progression surrogates of age (left plots) and months non-ambulatory (middle plots), as well as follow-up visit (right plots).

**Table 4:** Group mean (SD) of the percentage change from baseline for the summary measures of cross-sectional area (CSA), total distance of motion (TDM) of the diaphragm, and diaphragm length (DIA).

	Duchenne muscular dystrophy (DMD) visit 2 (3 months, *N* = 10)				DMD visit 3 (6 months, *N* = 10)				DMD visit 4 (12 months, *N* = 8)			
			
	Min	Max	Delta	Mean	Min	Max	Delta	Mean	Min	Max	Delta	Mean
CSA change (%)	−4.67 (9.5)	0.38 (16.7)	17.5 (53.8)	−0.02 (7.78)	0.81 (20.3)	1.44 (27.0)	17.6 (72.6)	4.66 (18.6)	−15.3 (19.3)*	−3.62 (25.2)	17.8 (56.1)	−6.53 (20.5)
TDM change (%)	−4.13 (6.7)	−1.77 (11.3)	12.4 (46.0)	−1.24 (7.8)	−0.18 (15.7)	−3.03 (14.6)	2.85 (57.5)	0.97 (11.6)	−10.8 (18.4)	−4.97 (14.7)	14.1 (40.7)	−6.56 (13.9)
DIA change (%)	−2.58 (7.3)	−0.41 (13.8)	10.1 (43.3)	−2.48 (10.0)	0.96 (10.5)	−1.27 (20.6)	−0.55 (55.1)	−2.88 (15.9)	0.49 (8.3)	1.83 (14.3)	7.63 (39.1)	0.58 (10.4)

*P ≤ 0.05 for post hoc pairwise comparisons of absolute measurements between visits, two-tailed.

The conventional measures of respiratory function, FVC sitting and %Pred, showed no significant longitudinal changes. However, there was a significant main effect of visit for both min CSA [*F*(3,13.21) = 6.16, *P* = 0.008] and min PST [*F*(3,16.28) = 5.45, *P* = 0.009], and pairwise comparisons between visits suggested that the 12-month measurements were significantly decreased compared to baseline [min CSA: −13.29 (95% confidence intervals: −23.62 to −2.96), *P* = 0.016; min PST: −12.81 (−23.74 to −1.88), *P* = 0.024—displayed as percentage change from baseline in Table 4 and Figure 6 right side plots]. The min TDM had a 10.8% decrease at 12 months compared to baseline (borderline significance in pairwise comparisons: −33.02 (−66.08 to 0.033), *P* = 0.050) and the max TDM was numerically reduced at each visit (Table 4), but these MRI summary measures did not display a main effect of visit in this relatively small cohort.

Several of the MRI-derived summary measures related to the disease progression markers of age and months without ambulation (Table 5). There were relationships of min CSA, min TDM, and min PST with both age and the number of non-ambulatory months (*P* ≤ 0.05 for all). In contrast, only the spirometry metrics of %Pred and MEP were related to the months of non-ambulation [*t* = −2.75, *P* = 0.012 (*N* = 31); *t* = 3.17, *P* = 0.004 (*N* = 34)], and the MEP related to age [*t* = 2.31, *P* = 0.032 (*N* = 33)].

**Table 5:** Table of *t*-statistics for the statistically significant relationships between the Magnetic Resonance Imaging summary measures and the disease progression markers of time since loss of ambulation (LOA) in months and age in years.

	DMD (all visits)						
	Min CSA	Min TDM	Max TDM	Min ANT	Max ANT	Mean ANT	Min PST
LOA in months (*N* = 37)	−2.22*	−2.08*	–	–	–	–	−2.31*
Age in years (*N* = 35)	−2.13*	−2.44*	−2.21*	−2.70**	−2.25*	−2.37*	−2.22*

**P ≤ 0.01, *P ≤ 0.05.

*DMD, Duchenne muscular dystrophy; CSA, cross-sectional area; TDM, total distance of motion*.

## Discussion

We have reported herein the respiratory profile of a cohort of glucocorticoids treated non-ambulant DMD subjects as recorded by spirometry, lung and diaphragm measures from dynamic MRI. The MRI data were compared at baseline to age- and gender-matched healthy controls. The novel semi-automated analysis pipeline developed for this study was used for calculation of temporal profiles of lung and diaphragm measures from dynamic MRI acquired in the sagittal imaging plane, and extraction of key summary measures from these temporal profiles. This work lays the foundations for a robust, fully automated computation tool for measures of respiratory function that may be more specific and sensitive to disease related changes in DMD than conventional spirometry.

At baseline, DMD subjects were not compromised from a respiratory function point of view with a mean (SD) spirometry measure of 2.09 (0.50) liters (L) for FVC, 71.2 (12.5) for %Pred, and 3.98 (1.06) L/min for PEF. Spirometry measurements in the DMD boys were overall stable over the course of study (12 months): −2.0% (17.0) and −6.4% (14.4) in FVC and %Pred, respectively, of no statistical significance. Literature pertaining to the natural history of DMD does suggest that by their mid-teens DMD boys will typically have some respiratory impairment and annual progression, and despite contemporary management with steroids and improved cardiac care, would be expected to require ventilation by the age of 16–20 years (20–22). It is, therefore, possible that some respiratory impairment and progression was present in our DMD cohort, but that our study was underpowered to detect this respiratory impairment/progression by spirometry due to the relatively small sample size. In support of respiratory function progression, the spirometry metrics of %Pred and MEP were related to the disease progression marker of months non-ambulation. No statistically significant difference was found between the baseline values of FVC sitting and FVC supine; however, in such a small sample, this standalone finding is insufficient to confirm their equivalence. The finding should, therefore, be taken together with literature reports of the equivalence of FVC between the two positions (18, 22).

In regards to MRI, we initially explored the linearity of chest wall motions in both health and disease, finding evidence of multiple components to diaphragm movement that are variably affected in DMD. Most of the derived MRI summary measures (that provide a statistical descriptive of the response to diaphragmatic motion) had highly significant baseline differences compared to controls and displayed good correlation with the baseline spirometry data (of FVC, %Pred, and PEF). We did not adjust for height in the baseline relationships between MRI and spirometry data; however, both MRI and spirometry measures are expected to be dependent upon height. Longitudinally, there was a significant decrease in min CSA at the last visit (12 months) and several of the MRI-derived summary measures (in particular: min CSA, min TDM, and min PST) related to the continuous metrics of disease progression/severity in DMD, while such findings in the spirometry data were limited (to relationships of %Pred and MEP with months of non-ambulation, and MEP with age). Since the heights of the DMD boys were stable over the study duration (12 months), the longitudinal MRI findings are not driven by patient size, and are in fact in opposition to findings expected from age-related size increases.

Previous studies exploring MRI measures of pulmonary function have focused on healthy volunteers (14–18). Cluzel et al. showed good agreement between spirometry and MR values of lung volume. Tomita et al. demonstrated that the sum of the three parameters of diaphragmatic motion [ANT, CNT, and PST as previously defined by Kondo et al. (16)] gives a good indication of the TDM of the diaphragm, and this measure does not significantly change between the supine and prone scanning positions. Kondo et al. measured lung CSA and 1D lung lengths in nine healthy volunteers, finding a linear correlation between lung volume (obtained from a pneumotachograph) and these measures. The slope of linear regressions were used to compare the contribution of the length and CSA measures to the change in lung volume, while the *y*-axis intercepts represented the length or CSA at end expiration.

Here, we used a similar regression analysis technique as Kondo et al. to instead explore the linearity of the change in length measures with change in lung CSA (16). Like Kondo et al., we found strong correlations between the variables. However, there was a consistently lower contribution to the CSA change (*S*) from the anterior part of the diaphragm (ANT) than the CNT and the PST contributions, emphasizing that the diaphragm does not move as one functional unit during deep breathing. There were significantly stronger correlations of CNT, TDM, and DIA among the healthy controls than the DMD subjects, suggesting a relative loss of synchrony in diaphragm movement during deep breathing with DMD. Moreover, we found that a larger change in the TDM of the diaphragm is required in DMD patients for a unit change in CSA, suggesting that the diaphragmatic motion is less efficient for changing lung capacity in DMD.

With regard to the MRI-derived summary measures of diaphragmatic motion, we hypothesize that the significant decrease in min CSA at 12 months reflects combined effects of weakening diaphragmatic and inter-costal muscles over the 1-year study period. In addition, the numerical decrease in max TDM across visits suggests progressive diaphragm dysfunction in this DMD cohort. The significant relationship of several of the summary measures with the disease progression markers of age and months without ambulation is particularly encouraging as it suggests that they provide a clinically meaningful measure of diaphragmatic function in DMD, and likewise for a subset of the spirometry measures.

One desirable feature of the summary measures is that they can be extracted from a subset of the dynamic MRI data—a collection of frames close to the maximal inspiration and expiration—opening-up both the option of shorter scan durations for patients who cannot tolerate time in the scanner and/or multiple deep breath cycles, and reduced computational time for the image processing. We should be mindful, however, that the min and max summary measures are extracted from single points on the temporal profiles of the lung CSA and length measures, so they have the potential to be susceptible to noise (spikes) on these profiles. Future extensions to this work could investigate this potential problem and appropriate avoidance methods (e.g., smoothing of the temporal profiles, or extraction of max and min summary measures from the average of multiple peaks or troughs on the temporal profiles, respectively), in addition to extending the analysis pipeline to other imaging planes.

Although spirometry is currently the most common test for pulmonary function in DMD, as an outcome measure in clinical trials it requires a large number of subjects to account for the wide variability. Indeed, the spirometry metrics showed no significant longitudinal changes in our study, despite expected progression of respiratory function in DMD boys of this age (20–22). Furthermore, measures can be heavily reliant on subject cooperation and motivation to perform the tests and, in DMD, comprehension and cognitive function. On the other hand, MRI appears to offer a sensitive and targeted measure of diaphragm function. While still requiring collaboration in relation to taking a breath in and out, and sitting/lying in the scanner, the maneuvers required for the MRI assessment are less dependent on coordination and following specific commands. The MRI parameters may also offer a more extensive measurement and more flexibility to probe specific, individual components of pulmonary function (such as the lower contribution from the anterior part of the diaphragm to the CSA change) since multiple imaging planes and targeted regions-of-interest, on a slice-by-slice or volumetric basis, can be objectively explored with relative ease and accuracy.

In summary, through the development of a novel image analysis pipeline, this work offers an efficient method of extracting lung and diaphragm measures from dynamic MRI and expands our understanding of the natural history of respiratory function in DMD; suggesting that MRI could provide an adjunctive and sensitive/objective measure of pulmonary function in DMD. This is of particular importance and relevance because some promising novel pharmaco–gene therapies have shown very good uptake in the diaphragm (23–25), so MRI-derived measures of respiratory function and diaphragm mobility could be suitable objective outcome measures in clinical trials of such experimental therapies, as well as allowing non-ambulant boys to be followed-up in future studies. We suggest that future studies with larger sample sizes are performed to confirm the proposed utility of MRI for assessing lung respiratory function in DMD patients.

## Ethics Statement

Approval from the Brighton & Sussex Research National Ethics Committee was obtained for this study, which was performed in compliance with the Declaration of Helsinki. Written informed consent for participation in the study was obtained from a representative parent or caregiver and assent forms were signed by the DMD boys and healthy volunteers.

## Author Contributions

CB was responsible for the analysis and interpretation of data, the writing, revision, and final approval of the manuscript. VR contributed to the study design, was responsible for subject recruitment and clinical assessments, as well as the critique, writing and approval of the manuscript. CS, ME, JB, JM, MH, and JT contributed to data acquisition, critique, and approval of the manuscript. PM, TY, FM, and RJ were responsible for the conception and initial design of this study, manuscript design, critique, and approval. FM additionally secured the funding from GSK for this study. RN contributed to data interpretation, critique, and revision of the manuscript, as well as final approval.

## Conflict of Interest Statement

CB is an employee of Imanova Limited. VR is currently an employee of Solid Biosciences. RJ is an employee of GlaxoSmithKline. PM was an employee of GlaxoSmithKline during design and initial implementation stages of this study. TY has received honoraria and travel expenses for advisory committee work from Bayer Schering, Biogen Idec, and Novartis; and research grants (held by University College London) from Biogen Idec, GlaxoSmithKline, Novartis, and Schering AG for analysis of data from MS trials. JT has received research support from GlaxoSmithKline, Medtronic, and Siemens. FM has served on scientific advisory boards for AcceleronPharma, Genzyme, Sarepta Therapeutics, Debiopharma Group, GlaxoSmithKline, Prosensa, Servier, Summit, Capricor, Italfarmaco, Pfizer, and Santhera Pharmaceutical, and UCL receives research support from Roche, Summit, Sarepta, PTC Therapeutics and GlaxoSmithKline. All other authors declare that the research was conducted in the absence of any commercial or financial relationships that could be construed as a potential conflict of interest. The reviewer AT and handling editor declared their shared affiliation.

## Supplementary Material

Data Sheet S1 A text file providing details of the image analysis pipeline as well as the results of the intra- and inter-observer variability testing of the analysis method.

Figure S1 A Schematic representation of the image analysis pipeline, including the key image processing steps and the generated outputs.

Figure S2 The min ANT (top left), min CNT (top right), min PST (bottom left), and min DIA lengths (bottom right) computed from the new sets of initialization points provided by the two observers.

Video S1 A video showing the lung segmentation and the length measures (ANT, CNT, PST, DIA) on every frame of a sample dynamic MRI.
