# Supplementary material for: Semi-Automated Analysis of Diaphragmatic Motion with Dynamic Magnetic Resonance Imaging in Healthy Controls and Non-Ambulant Subjects with Duchenne Muscular Dystrophy
Source: Front Neurol. 2018 Jan 26;9:9. doi: 10.3389/fneur.2018.00009 (PMC5790781; doi:10.3389/fneur.2018.00009)
Supplement: Data Sheet S1 — A text file providing details of the image analysis pipeline as well as the results of the intra- and inter-observer variability testing of the analysis method. [file data_sheet_1.docx]

Supplementary Material

Semi-automated analysis of diaphragmatic motion with dynamic MRI in healthy controls and non-ambulant subjects with Duchenne muscular dystrophy

Courtney A. Bishop*, Valeria Ricotti, Christopher DJ Sinclair, RB Matt Evans, Jordan W Butler, Jasper M Morrow, Michael G Hanna, Paul M Matthews, Tarek A Yousry, Francesco Muntoni, John S Thornton, Rexford D Newbould, Robert L Janiczek

*** Correspondence:** Courtney A. Bishop: courtney.bishop@imanova.co.uk

# The Image Analysis Pipeline

An analysis pipeline was developed to extract temporally-varying profiles of lung and diaphragm measures from dynamic MRI data for the investigation of exploratory endpoints related to pulmonary function. S1 Supplementary Figure illustrates the image analysis pipeline and the generated outputs, including the lung and diaphragm summary measures. After loading the deep-breathing sagittal-plane dynamic MRI data into MATLAB (http://uk.mathworks.com/products/matlab/), the semi-automated analysis pipeline includes the following key steps to arrive at the extracted lung and diaphragm measures:

- Body Contouring – initialises a contour at the edge of the image and uses an active contouring approach to direct this contour inwards to find the high intensity boundary at the edge of the body, separating/extracting the body from the image background on each and every frame of the dynamic MRI (note: image de-noising also performed at this stage using median filtering)
- Airway Definition – finds low intensity regions inside the body using a simple intensity thresholding approach on each image frame (note: default threshold is 25th percentile of maximum intensity in the de-noised image, but the user can specify an alternative threshold at start-up if required)
- Lung Initialization – uses morphological refinement (filling of holes and closing of gaps between isolated, low-intensity structures) followed by a connected component analysis to find connected regions in image space, then selects the largest region for initialization of the lung segmentation on each image frame (note 1: if after morphological refinement only a single region - the lungs – exists, no region selection per se is required at the subsequent connected component analysis, but it serves a purpose if there are isolated pockets of other low intensity regions in the image, with the largest connected component corresponding to the lungs; note 2: the area chosen for lung initialization – the “initial lung label” - is also used for removal of hyper-intensities within the lung by first eroding this initial lung label and then assigning any hyper-intense pixels within (>90th percentile of values within the eroded lung region) to more “normal” (50th percentile) values, so that hyper-intense voxels do not interfere with the lung refinement step to follow).
- Lung Refinement – refines the initial lung contour to fit the boundaries of the lung on each frame of the dynamic MRI (similar to the Body Contouring step described above, but now performed within the bounds of the body to segment the lungs), using active contouring then morphological operations to close and fill any holes in the refined lung label (note: default is to perform morphological operations on the refined lung label but the user can switch off this option if preferred)
- Lung CSA Measurement – calculates the full lung cross-sectional area (CSA) on each sagittal frame of the dynamic MRI as the area inside the refined lung contour.
- Landmark Initialization – asks the user to manually define three initialization points (seed points) outside the lung contour on the single frame with maximum CSA, proximal to the desired anatomical landmarks – the apex of the lung, and the edge of the diaphragmatic dome at the anterior and posterior chest wall margins (importantly, this is the only step of the analysis pipeline requiring manual input)
- Landmark Definition – automatically computes the anatomical landmarks on each and every frame of the dynamic MRI data based on the closest Euclidean distance from the three initialization points to the lung boundary contour
- Length Measurement – automatically extracts the parameters of diaphragmatic motion (the 1D lung lengths: ANT, CNT and PST) and their sum (TDM), as well as the full diaphragm length (DIA), on each and every frame of the dynamic MRI data.

# Intra- and inter-observer variability

For the min ANT, min CNT, min PST, and min DIA lengths calculated from the sets of user initialization points, the percentage coefficients of variation were calculated for each individual observer and each test subject. The results are given in the following tables:

|  | **Test Subject** | | | | | |  |
| --- | --- | --- | --- | --- | --- | --- | --- |
| **Observer** | DMD-1 | DMD-2 | DMD-3 | CON-1 | CON-2 | CON-3 |  |
| CB | 0 | 0 | 0.841 | 0.002 | 0 | 0.062 | 0.151 |
| GS | 0.208 | <0.001 | 0.010 | 0.002 | 0 | 0 | 0.037 |
|  | 0.104 | <0.001 | 0.425 | 0.002 | 0 | 0.031 |  |

Supplementary Table 2.1. %CV for Min ANT Length (mm)

|  | **Test Subject** | | | | | |  |
| --- | --- | --- | --- | --- | --- | --- | --- |
| **Observer** | DMD-1 | DMD-2 | DMD-3 | CON-1 | CON-2 | CON-3 |  |
| CB | 0.739 | 0 | 1.478 | 0.139 | 0.182 | 0.093 | 0.438 |
| GS | 2.504 | 1.187 | 0.844 | 0.568 | 0.222 | 0 | 0.888 |
|  | 1.622 | 0.594 | 1.161 | 0.353 | 0.202 | 0.046 |  |

Supplementary Table 2.2. %CV for Min CNT Length (mm)

|  | **Test Subject** | | | | | |  |
| --- | --- | --- | --- | --- | --- | --- | --- |
| **Observer** | DMD-1 | DMD-2 | DMD-3 | CON-1 | CON-2 | CON-3 |  |
| CB | 0.502 | <0.001 | 0 | <0.001 | 0.093 | 0.059 | 0.109 |
| GS | 0.502 | 0.003 | 0.003 | 0.003 | 0.093 | 0 | 0.101 |
|  | 0.502 | 0.002 | 0.001 | 0.002 | 0.093 | 0.029 |  |

Supplementary Table 2.3. %CV for Min PST Length (mm)

|  | **Test Subject** | | | | | |  |
| --- | --- | --- | --- | --- | --- | --- | --- |
| **Observer** | DMD-1 | DMD-2 | DMD-3 | CON-1 | CON-2 | CON-3 |  |
| CB | 0.001 | 0.149 | 0 | 0 | 0.065 | 0 | 0.036 |
| GS | 0.613 | 0.162 | 0 | 0 | 0.614 | 0 | 0.232 |
|  | 0.307 | 0.155 | 0 | 0 | 0.340 | 0 |  |

Supplementary Table 2.4. %CV for Min DIA Length (mm)
