# Supplementary material for: Semi-Automated Analysis of Diaphragmatic Motion with Dynamic Magnetic Resonance Imaging in Healthy Controls and Non-Ambulant Subjects with Duchenne Muscular Dystrophy
Source: Front Neurol. 2018 Jan 26;9:9. doi: 10.3389/fneur.2018.00009 (PMC5790781; doi:10.3389/fneur.2018.00009)
Supplement: Figure S1 — A Schematic representation of the image analysis pipeline, including the key image processing steps and the generated outputs. [file image_1.PDF]

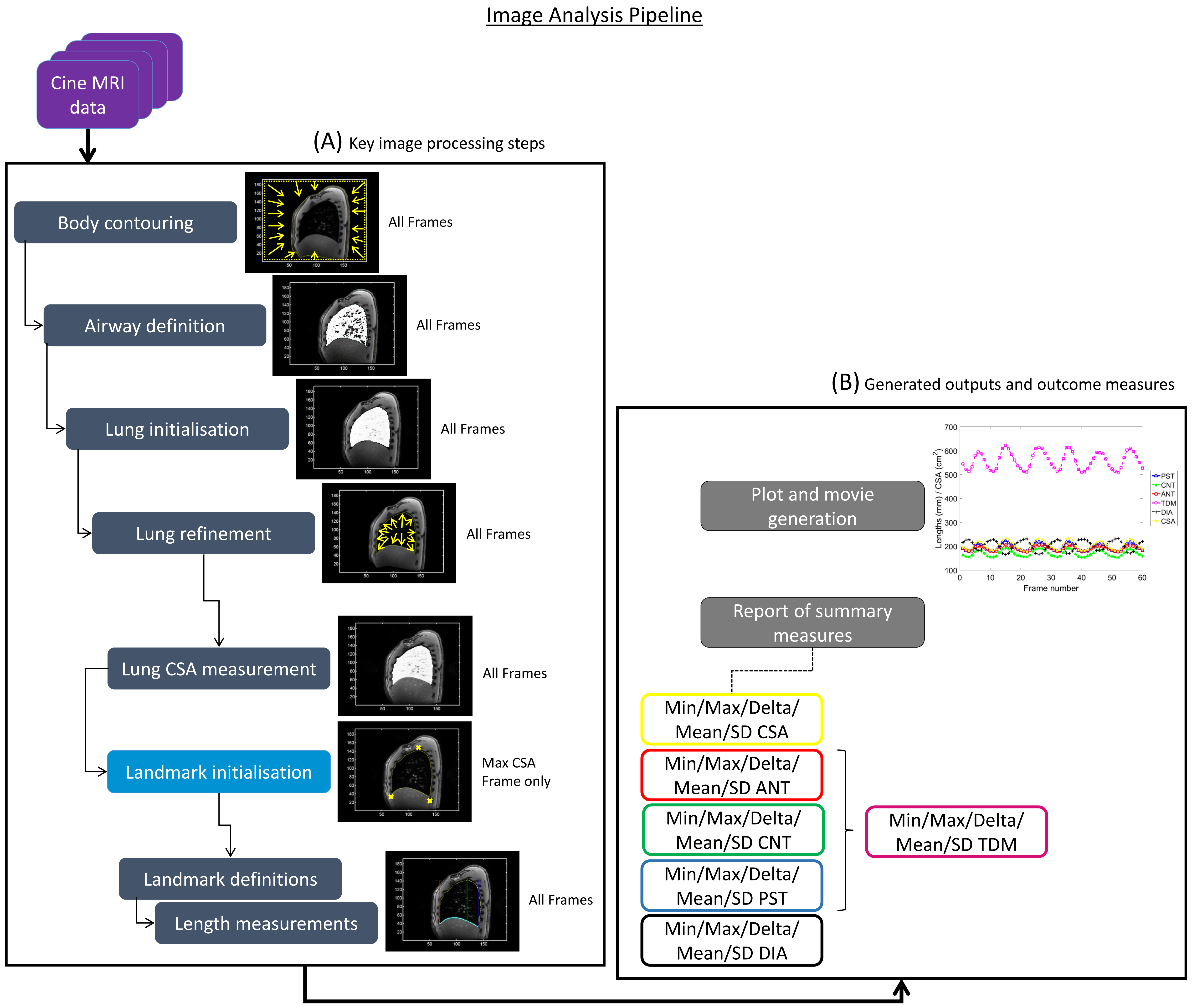

Supplementary Figure 1. A schematic of the image analysis pipeline developed in MATLAB, including the key image processing steps (A) and the generated outputs (B) which contain a plot of the temporal profiles of the lung cross-sectional area (CSA), the lung 1-D length measures (ANT, CNT and PST) and their sum (TDM), as well as the full diaphragm length (DIA).
