# Supplementary figures and images for: Semi-Automated Analysis of Diaphragmatic Motion with Dynamic Magnetic Resonance Imaging in Healthy Controls and Non-Ambulant Subjects with Duchenne Muscular Dystrophy
Source: Front Neurol. 2018 Jan 26;9:9. doi: 10.3389/fneur.2018.00009 (PMC5790781; doi:10.3389/fneur.2018.00009)

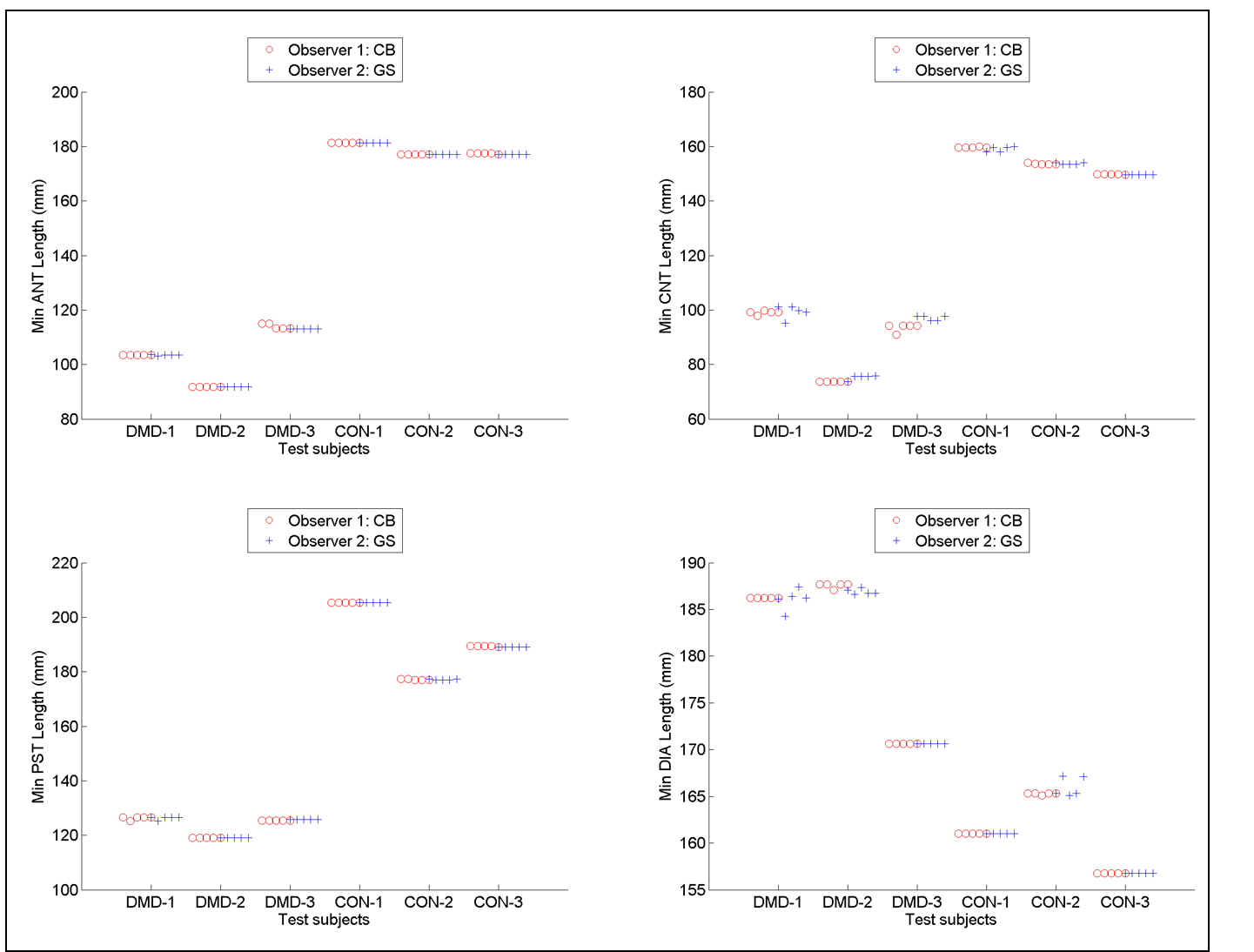

Supplement: Figure S2 — The min ANT (top left), min CNT (top right), min PST (bottom left), and min DIA lengths (bottom right) computed from the new sets of initialization points provided by the two observers. [file image_2.tif]
